# Biomarkers of progressive multiple sclerosis decrease following autologous hematopoietic stem cell transplantation

**DOI:** 10.1186/s12974-025-03511-6

**Published:** 2025-07-17

**Authors:** Ida Erngren, Katarina Lundblad, Ivan Pavlovic, Asma Al-Grety, Anders Larsson, Kim Kultima, Joachim Burman

**Affiliations:** 1 Department of Medical Sciences, Clinical Chemistry, Uppsala University, Uppsala, Sweden; 2 Department of Medical Sciences, Translational Neurology, Uppsala University, Uppsala, Sweden

**Keywords:** Multiple sclerosis, CSF, Autologous hematopoietic stem cell transplantation, Biomarker, YKL-40, GDF-15, Gal-9

## Abstract

**Background:**

Autologous hematopoietic stem cell transplantation (AHSCT) has been increasingly used for treatment of relapsing-remitting multiple sclerosis (RRMS). Existing data suggest that AHSCT might alter the natural course of multiple sclerosis (MS) and postpone or even prevent the occurrence of progressive MS. This study aimed to investigate whether three cerebrospinal fluid biomarkers of progressive MS: Galectin-9, GDF-15, and YKL-40, were affected by treatment intervention with AHSCT for RRMS.

**Methods:**

RRMS patients treated with AHSCT at Uppsala University Hospital between 2011 and 2018 were considered for participation and included if CSF samples from baseline and at least one follow-up were available. CSF from healthy volunteers was included as controls. Galectin-9 and GDF-15 concentrations were determined with ELISA, and YKL-40 with electrochemiluminescence.

**Results:**

The final cohort comprised 45 RRMS patients and 32 controls. At baseline, MS patients had markedly higher CSF concentrations of Galectin-9 and YKL-40 and slightly higher GDF-15 than controls. Following AHSCT, biomarker concentrations decreased from baseline to the 1-year follow-up, with a median (IQR) of 454 (357–553) vs. 408 (328–495) pg/mL (*P* = 0.0002) for Galectin-9; 49 (38–79) vs. 45 (35 to 75) pg/mL (*P* = 0.012) for GDF-15, and 100 (54–164) vs. 58 (43–92) ng/mL (*P* < 0.0001) for YKL-40. Galectin-9 and YKL-40 concentrations decreased further and were even lower at the 2-year follow-up; median (IQR) 408 (328–495) vs. 376 (289–478) pg/mL (*P* = 0.0009) for Galectin-9; and 62 (37–96) vs. 56 (30–83) ng/mL (*P* < 0.0001) for YKL-40. Thereafter, the levels of all biomarkers were stable throughout the follow-up.

**Conclusion:**

Treatment with AHSCT was associated with sustained reductions in biomarkers linked to progressive MS, indicating its potential not only to achieve lasting remission but also to delay or prevent transition to SPMS. However, additional studies are necessary to confirm these findings and elucidate their long-term clinical significance.

**Supplementary Information:**

The online version contains supplementary material available at 10.1186/s12974-025-03511-6.

## Background

In recent years, autologous hematopoietic stem cell transplantation (AHSCT) has become increasingly recognized as a viable treatment option for severe multiple sclerosis (MS) of the relapsing-remitting type (RRMS) [1, 2]. It is a one-time procedure that targets disease-promoting immune cells to attain permanent and beneficial changes in the immune system. Most patients experience long-lasting remission following AHSCT [3], and the outcome of AHSCT has repeatedly been shown to compare favorably with that of conventional disease-modifying drugs (DMDs) [4, 5].

It is less clear if AHSCT affects the biological processes associated with progressive MS and if the transition from RRMS to secondary progressive MS (SPMS) can be postponed or even avoided altogether. It has previously been reported that aberrantly activated microglia and astrocytes and escalating mitochondrial damage are key elements of progressive MS development [6, 7]. Biomarkers reflecting these processes are hence of interest when probing whether AHSCT affects the transition to SPMS.

Galectin-9 (Gal-9) belongs to the galectin family of carbohydrate-binding proteins, with their shared affinity for the basic carbohydrate unit galactose [8, 9]. Through interaction with the T-cell immunoglobulin mucin 3 (TIM-3), Gal-9 inhibits differentiation of naïve T-cells into T-helper 17 cells (Th17) [10], causes apoptosis of mature Th17 and T-helper 1 (Th1) cells, and promotes Foxp3^+^ regulatory T cells [11, 12]. In contrast, Gal-9 has a proinflammatory effect on the innate immune system [13]. Gal-9 also has a vital role in the astrocyte-microglia-activation cycle where it stimulates microglial production of TNF-α and Il-6 [14], and TNF-α does, in turn, stimulate Gal-9 production by astrocytes [15]. Increased levels of Gal-9 have also been associated with progressive MS [16].

Growth differentiation factor 15 (GDF-15) belongs to the TGF-β superfamily and is a stress-responsive cytokine that regulates several physiological and pathological processes [17, 18]. GDF-15 has been proposed as a biomarker for diagnosis, prognosis, and/or risk stratification of various patient populations, such as cardiovascular disease, kidney disease, liver disease, metabolic syndrome, diabetes mellitus, and sepsis, to mention a few [19]. Increased serum levels of GDF-15 have also been associated with neurodegenerative diseases [20], and increased CSF levels have been demonstrated in patients with progressive MS [21].

YKL-40, also known as chitinase 3-like protein 1 (CHI3L1), belongs to the chitinase-like glycoprotein family. Within the CNS, YKL-40 is predominantly produced by activated astrocytes but also, to some extent, by microglia [22, 23]. The physiological function of YKL-40 is, for the most part, unknown, though a role in tissue remodeling in connection to inflammation has been suggested [22]. Several studies have found elevated YKL-40 concentrations in the CSF of patients with clinically isolated syndrome (CIS) and MS [24]. In a histopathological study, YKL-40 was expressed by microglia as well as by activated astrocytes at the rim of chronically active lesions [23]. High CSF levels of YKL-40 in patients with progressive MS have been reported previously [25, 26] and have also been associated with worse prognosis [25, 27].

The current study aimed to investigate if intervention with AHSCT was associated with a change in the CSF concentrations of Gal-9, GDF-15, and YKL-40 in a cohort of RRMS patients.

## Methods

### Subjects

A total of 85 patients diagnosed with RRMS according to the revised McDonald criteria [28] and treated with AHSCT at Uppsala University Hospital in the time period January 1st, 2011, to December 31st, 2018, were screened for participation. Patients were included if CSF samples from baseline and at least one follow-up were available on June 30th, 2020. A control group of 32 volunteers without neurologic disease was included as a reference.

### Procedures

Autologous hematopoietic stem cells were mobilized with a single dose of 2 g/m^2^ cyclophosphamide followed by filgrastim 5–10 µg/kg/day for 6–7 days and then harvested approximately 10 days after the start of the mobilization regimen. No ex-vivo graft manipulation was performed. Patients were conditioned with a combination of cyclophosphamide and rabbit anti-thymocyte globulin (cyclophosphamide 200 mg/kg; rATG 6 mg/kg). After conditioning, autologous hematopoietic stem cells were thawed and reinfused. Prophylaxis for fungal, viral, and bacterial infection was administrated during neutropenia. Prophylaxis for herpes viruses and *Pneumocystis jiroveci* continued for a minimum of 3 months.

### Lumbar punctures, CSF handling, and storage

Lumbar punctures were made at baseline and routinely at follow-up at 1, 2, and 5 years. In a few cases, follow-up with lumbar puncture was postponed, and in some instances, additional lumbar punctures were made. Not all patients had completed their 5-year follow-up; in such instances, only samples collected at earlier timepoints were included in the analysis. The controls underwent lumbar puncture at a single time point. CSF samples were handled according to a 2009 consensus protocol on CSF biobanking [29].

### Quantification of Gal-9, GDF-15, and YKL-40 in CSF

Gal-9 was analyzed with a Human Galectin-9 DuoSet ELISA (R&D Systems, Minneapolis, MN, United States. Cat. No. DY2045), and GDF-15 was analyzed with a Human GDF-15 DuoSet ELISA (R&D Systems, Minneapolis, MN, United States. Cat. No. DY957) in the same laboratory by board certified technicians who were blinded to clinical data. YKL-40 was analyzed with electrochemiluminescence at the Science for Life facility in Uppsala, using a multiplex proteomics platform: the Meso Scale Discovery (MSD) U-PLEX™ Metabolic Group 1 Assay (Meso Scale Discovery, Gaithersburg, MD, USA. Cat. No. K15280K). The intra-assay CV (pooled CV for all plates) was 2.8% for Gal-9, 3.2% for GDF-15, and 2.6% for YKL-40.

### Definitions

A *clinical relapse* was defined as a period of acute worsening of neurological function lasting ≥ 24 h and not attributable to an external cause such as increased body temperature or acute infection. *Confirmed disability worsening* (CDW) was defined as an increase in EDSS score with at least one point from baseline, sustained between two follow-up visits separated in time by no less than 6 months (1.5 points if EDSS at baseline was 0; 0.5 points if the baseline EDSS ≥ 5.5). An *MRI event* was defined as the appearance of any T2 lesion > 3 mm or gadolinium-enhancing lesion in the brain or spinal cord not present on the baseline scan. *Active disease* was defined as the presence of a clinical relapse *or* at least one gadolinium-enhancing lesion ± 1 month from the lumbar puncture. *No evidence of disease activity* (NEDA-3) was defined as the absence of clinical relapses, CDW, and MRI events. Patients who did not maintain NEDA-3 were considered to have *evidence of disease activity* (EDA). Dimethyl fumarate, fingolimod, glatiramer acetate, and interferons were considered to be *1st line* treatments, whereas natalizumab and rituximab were considered to be *2nd line* treatments.

### Statistical analyses

Data were summarized as medians with interquartile ranges (IQR). Correlations were described using Spearman’s r. Comparisons between two groups were analyzed with the Mann-Whitney test for unpaired data and the Wilcoxon signed-rank test for paired data. For comparisons involving more than two unpaired groups, the Kruskal-Wallis test was employed, followed by Dunn’s multiple comparisons test. Multiple linear regression was used to assess potential confounding effects of age and sex; biomarker values were adjusted accordingly if a statistically significant effect was identified. Statistical significance was defined as a two-tailed *p*-value < 0.05. Correlation analyses were adjusted for multiple comparisons using the Benjamini-Hochberg procedure.

## Results

The final study comprised 29 females and 16 males with RRMS, aged 19–46 at baseline, and 15 females and 17 males, aged 18–48, in the control group (Table 1). Within the primary cohort, 135 CSF samples were analyzed: 45 baseline samples and 90 post-AHSCT samples. Baseline samples were obtained at a median (IQR) of 1 (0–11) day(s) before the mobilization of stem cells and 40 (38–56) days before reinfusion of hematopoietic stem cells. Follow-up samples were taken at 1-year follow-up (*n* = 44), 2-year follow-up (*n* = 32), 3-year follow-up (*n* = 3), 4-year follow-up (*n* = 1), and 5-year follow-up (*n* = 10). The 32 controls underwent lumbar puncture at a single time point only.

Most patients had been treated with DMDs at some time point before AHSCT, and only five patients were DMD naïve. At baseline, ten were not currently receiving DMD treatment, 14 were treated with orals and/or injectables, and 21 with natalizumab or rituximab.

**Table 1:** Demographic summary

	Baseline	1 year	2 years	3 years	4 years	5 years	Controls
N	45	44	32	3	1	10	32
Female/Male	29/16	28/16	23/9	2/1	1/0	7/3	15/17
Age, y, median (IQR)	30 (26–34)	31 (27–36)	33 (28–39)	29 (27–42)	30 (30–30)	36.5 (31–41)	22 (21–26)
Disease duration, y, median (IQR)	4.3 (1.9–9.4)	5.3 (2.9–10)	6.4 (4.9–11)	12 (6.0–14)	6.8 (6.8–6.8)	9.8 (7.1–14)	NA
Active disease during follow-up, n (%)	15 (33)	0 (0.0)	1 (3.1)	0 (0.0)	0 (0.0)	1 (10)	NA
DMD at baseline, n (%)	35 (78)	NA	NA	NA	NA	NA	NA

### Influence of age and sex

**Fig. 1:**
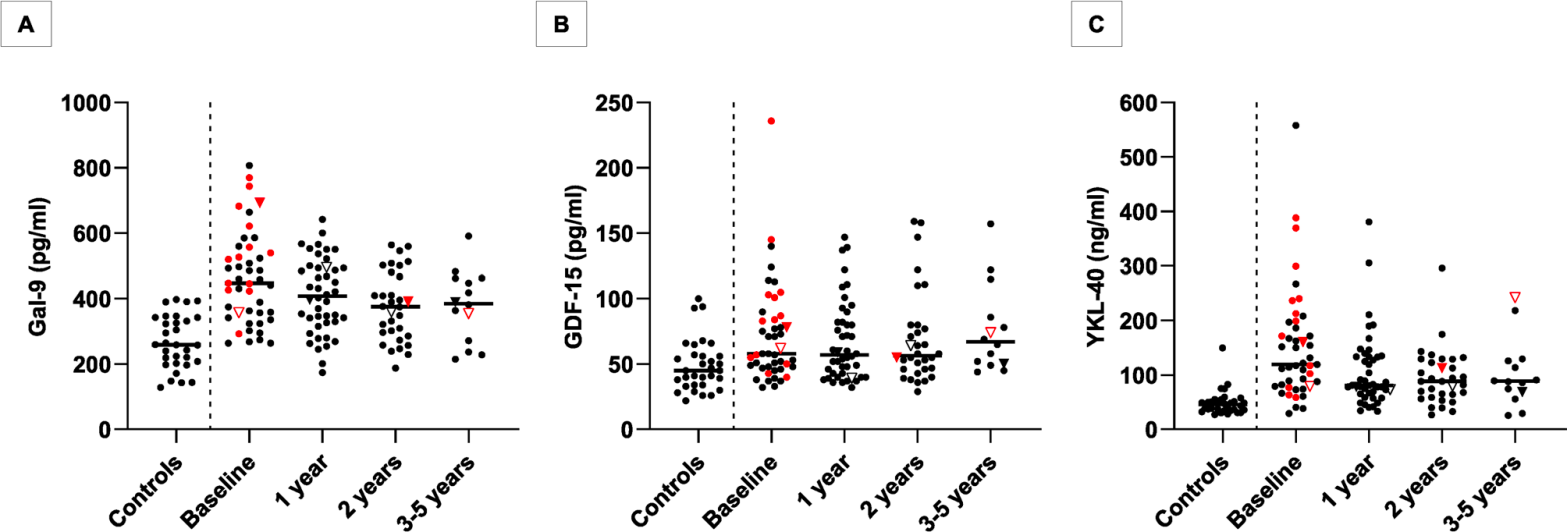
Overview of Gal-9, GDF-15, and YKL-40 concentrations in CSF from patients and controls. Overview of Gal-9 (**A**), GDF-15 (**B**), and YKL-40 (**C**) concentrations in cerebrospinal fluid of healthy controls without neurologic disease and patients with RRMS undergoing AHSCT. A vertical dashed line separates the controls from the patients. Patients were followed with repeated lumbar punctures for up to five years after AHSCT. Median values are highlighted by horizontal lines. Active disease (a clinical relapse or gadolinium-enhancing lesions on MRI) at the time when the sample was taken is denoted by red color. Two patients had a clinical relapse in conjunction with a follow-up visit; these patients are represented by triangles (one filled, one open). Note that this overview presents raw data only, *without* any adjustments for age

GDF-15 and YKL-40 were associated with age, whereas Gal-9 was not. Sex had no significant impact. Henceforth, GDF-15 and YKL-40 were adjusted for age. An overview of the unadjusted results is provided in Fig. 1.

### Correlations between CSF biomarkers and routine CSF analyses

**Fig. 2:**
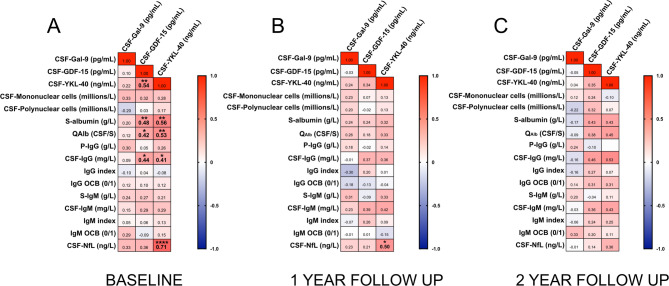
Correlation matrices of CSF Gal-9, GDF-15, YKL-40 and routine CSF analyses. Correlations are described with Spearman’s r and adjusted for multiple comparisons using the Benjamini-Hochberg procedure. At baseline, several correlations between Gal-9, GDF-15, YKL-40, and routine CSF analyses could be detected. Over time, these correlations were attenuated to the point that no statistically significant correlations could be seen. * *p* ≤ 0.05, ** ≤ 0.01

We then assessed correlations between the three biomarkers and standard CSF measurements, presenting these results as a correlation matrix (Fig. 2A). At baseline, GDF-15 showed a moderate correlation with YKL-40, whereas Gal-9 did not correlate significantly with either biomarker. Additionally, both GDF-15 and YKL-40 correlated moderately with serum albumin, the CSF/serum albumin quotient (QAlb), and CSF-IgG. YKL-40 also demonstrated a strong correlation with CSF-Neurofilament Light. At one year, only a moderate correlation between YKL-40 and CSF-Neurofilament Light persisted, and by two years, no statistically significant correlations remained (Fig. 2B-C).

### MS patients had higher CSF concentrations of Galectin-9, GDF-15, and YKL-40 than healthy controls

At baseline, MS patients had significantly higher CSF concentrations of Gal-9, GDF-15, and YKL-40 than healthy controls. The median (IQR) concentrations were 448 (350–548) vs. 259 (202–343) pg/mL (*P* < 0.0001) for Gal-9, 49 (39–78) vs. 43 (31–55) pg/mL (*P* = 0.042) for GDF-15, and 102 (55–166) vs. 41 (33–57) ng/mL (*P* < 0.0001) for YKL-40.

### MS patients with active disease had similar Galectin-9, GDF-15, and YKL-40 levels as those without active disease

**Fig. 3:**
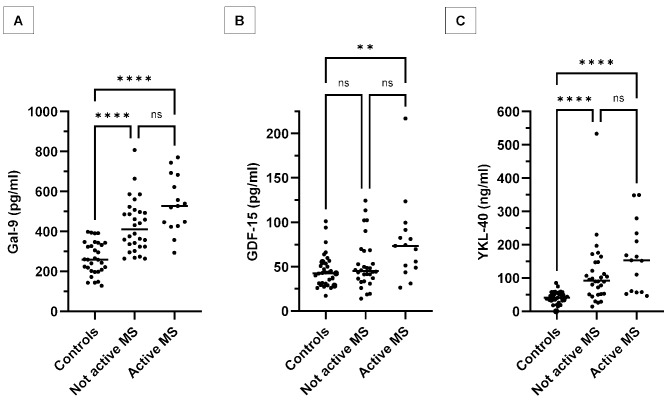
Comparisons of CSF Gal-9, GDF-15, and YKL-40 concentrations by disease activity status. Cerebrospinal fluid concentrations of Gal-9 (**A**), GDF-15 (**B**), and YKL-40 (**C**) in healthy controls without neurologic disease compared with baseline samples from patients with active / not active RRMS. Median values are highlighted by horizontal lines. ns = not significant, * *p* ≤ 0.05, ** ≤ 0.01, *** ≤ 0.001, **** *p* ≤ 0.0001

To assess whether patients with active disease at baseline had higher concentrations of Gal-9, GDF-15, and/or YKL-40 than those without, a three-way comparison was made between patients with active/not active disease and healthy controls (Fig. 3). There was no statistically significant difference in the levels of Gal-9/GDF-15/YKL-40 between the two groups. Patients with active disease vs. patients without, median (IQR): Gal-9 527 (427–683) vs. 410 (324–500) pg/mL (*P* = 0.19); GDF-15 73 (49–91) vs. 45 (36–68) pg/mL (*P* = 0.081); YKL-40 153 (58–234) vs. 92 (52–147) ng/mL (*P* = 0.41). There were no patients with active disease at 1-year follow-up, one patient with active disease at the 2-year follow-up and another at the 5-year follow-up (Table 1), which prohibited statistical analyses of comparisons at later time-points.

### Baseline treatment had no significant impact on Gal-9 and YKL-40 levels; whereas GDF-15 levels were lower in 2nd line DMD-treated than in untreated patients

To assess the influence of treatment with DMDs on the concentrations of Gal-9, GDF-15, and YKL-40, patients were separated into three groups according to their treatment status at baseline: untreated, 1st line treatment, and 2nd line treatment. Patients with 2nd line treatment had lower GDF-15 concentration in CSF than patients with 1st line treatment, with a median (IQR) of 44 (35–53) vs. 78 (53–102) pg/mL (*P* = 0.010). No other statistically significant results were detected (Supplementary Figure S1).

### Treatment with AHSCT was associated with a gradual decrease in both Galectin-9 and YKL-40 levels

**Fig. 4:**
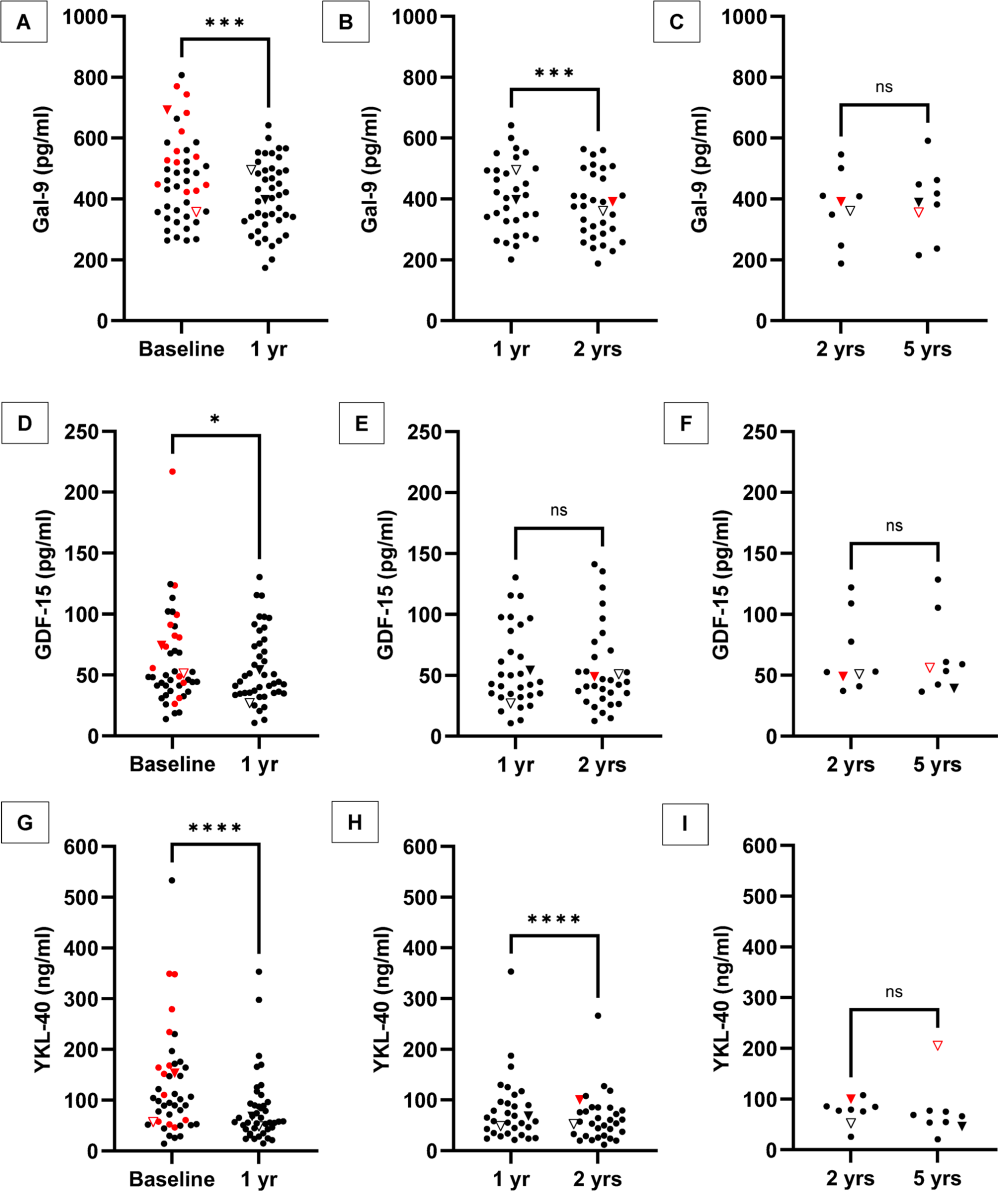
Comparisons of CSF concentrations of Gal-9, GDF-15 and YKL-40 before and after treatment with AHSCT. Cerebrospinal fluid concentrations of Gal-9 at (**A**) baseline vs. 1 year (*n* = 44), (**B**) 1 year vs. 2 years (*n* = 32 pairs), (**C**) 2 years vs. 5 years (*n* = 9); GDF-15 at (**D**) baseline vs. 1 year (*n* = 44), (**E**) 1 year vs. 2 years (*n* = 31 pairs), (**F**) 2 years vs. 5 years (*n* = 9); and YKL-40 at (**G**) baseline vs. 1 year (*n* = 44), (**H**) 1 year vs. 2 years (*n* = 32 pairs), (**I**) 2 years vs. 5 years (*n* = 9). Patients with active disease (a clinical relapse or gadolinium-enhancing lesions on MRI) at the time when the sample was taken are denoted by red color. Two patients had a clinical relapse in conjunction with a follow-up visit; these patients are represented by triangles (one filled, one open). ns = not significant, * *p* ≤ 0.05, ** ≤ 0.01, *** ≤ 0.001, **** *p* ≤ 0.0001

Following AHSCT, the CSF concentrations of Gal-9 and YKL-40 decreased from baseline to the 1-year follow-up, with a median (IQR) of 454 (357–553) vs. 408 (328–495) pg/mL (*P* = 0.0002) for Galectin-9; and 100 (54–164) vs. 58 (43–92) ng/mL (*P* < 0.0001) for YKL-40. Galectin-9 and YKL-40 concentrations decreased further and were even lower at the 2-year follow-up; median (IQR) 408 (328–495) vs. 376 (289–478) pg/mL (*P* = 0.0009) for Galectin-9; and 62 (37–96) vs. 56 (30–83) ng/mL (*P* < 0.0001) for YKL-40. The levels were stable throughout the 5-year follow-up (Fig. 4A–C and G–I).

### Treatment with AHSCT was associated with an initial slight decrease in GDF-15

Following AHSCT, the CSF concentrations of GDF-15 decreased slightly to the first follow-up at 1-year, median (IQR) 49 (38–79) vs. 45 (35 to 75) pg/mL (*P* = 0.012). GDF-15 concentrations were then stable throughout the follow-up period (Fig. 4D–F).

### Baseline levels of Gal-9, GDF-15, and YKL-40 were statistically unrelated to post-AHSCT EDA

To assess whether baseline concentrations of Gal-9, GDF-15, and YKL-40 could be determinants for EDA during the follow-up period after AHSCT, patients with at least 4 years of follow-up (*n* = 26) were considered. The patients were divided into two groups based on their NEDA/EDA status after AHSCT. For all three biomarkers, there were no statistically significant differences between the two groups (Supplementary Figure S2).

### Patients with post-AHSCT clinical relapse

Four patients had a clinical relapse during the follow-up period. Two of them also had active disease at one follow-up visit, and, in both cases, the YKL-40 level had risen before the relapse, whereas Gal-9 and GDF-15 concentrations were unaffected (Fig. 4).

One was clinically in remission at the time of the 2-year follow-up but had active disease on MRI. Sixteen days later, she experienced a clinical relapse, which prompted treatment with rituximab every six months for the rest of the follow-up period. Her YKL-40 levels were 153–68– 100–46 ng/mL (baseline, 1-year, 2-year and 5-year follow-up).

The other patient had a clinical relapse about two months after AHSCT. An MRI scan revealed one gadolinium-enhancing lesion in the spinal cord. Subsequent MRI scans did not show activity until the two-year follow-up, and no additional treatment was instigated. However, 6 months after the two-year follow-up, the patient had another clinical relapse with a new gadolinium-enhancing lesion in the spinal cord, prompting the start of rituximab treatment, with infusions every six months. Just before the 5-year follow-up, she had another clinical relapse despite treatment with rituximab. This time, the MRI scan showed no activity. The YKL-40 levels were 57–48– 52–205 ng/mL (baseline, 1-year, 2-year and 5-year follow-up).

## Discussion

This study demonstrates that CSF concentrations of Gal-9, GDF-15, and YKL-40 decrease significantly following AHSCT in patients with RRMS. Notably, the reductions in Gal-9 and YKL-40 were gradual and sustained over two years post-AHSCT. The decrease in GDF-15 was modest and stabilized within the first year. These findings provide important insights into the potential mechanisms by which AHSCT may influence the disease trajectory in RRMS, particularly concerning preventing or delaying SPMS development.

Elevated CSF concentrations of Gal-9 indicate ongoing astrocyte and microglia activation, which are key factors in the inflammatory processes underlying MS and the development of progressive MS [7, 14]. Gal-9 has a dual role depending on the disease stage: it may exert an immunoregulatory effect during RRMS, whereas in the context of SPMS, its elevated levels are likely associated with sustained CNS inflammation and disease progression [16]. Previous studies have reported higher CSF levels of Gal-9 in SPMS compared to RRMS, suggesting its potential as a biomarker for disease progression [16, 30]. This study showed a gradual decrease in Gal-9 levels over two years following AHSCT. This reduction may be attributed to decreased microglial activity, leading to lower TNF-α production and diminished astrocyte stimulation [14, 15]. These findings suggest that AHSCT may disrupt the astrocyte-microglia activation cycle, thereby mitigating the chronic compartmentalized CNS inflammation believed to contribute to progressive MS development. The observed decline in Gal-9 production is likely a consequence of the removal of activated immune cells through AHSCT. Notably, while Gal-9 plays a regulatory role in adaptive immunity [10–12], this reduction is unlikely to trigger post-AHSCT exacerbations. However, therapies that suppress Gal-9 levels without resetting or suppressing adaptive immunity, such as treatment with the TNF inhibitor lenercept, have been associated with MS exacerbations [31].

GDF-15 serves as a biomarker of mitochondrial dysfunction, a critical pathological mechanism underlying neurodegeneration in progressive MS. Mitochondrial dysfunction may result from inflammation driven by toxic reactive oxygen species and nitric oxide released by activated microglia, macrophages, and astrocytes [7, 32]. The link between GDF-15 and mitochondrial dysfunction highlights its potential as an indicator of disease progression and a target for therapeutic strategies aimed at mitigating neurodegeneration in advanced stages of MS [33–35]. We observed a modest reduction in CSF GDF-15 levels one year after AHSCT, which may indicate partial recovery from mitochondrial dysfunction. However, it is important to note that while AHSCT is unlikely to reverse existing mitochondrial damage, it may help prevent further damage driven by inflammation. In line with this, a previous study reported no change in CSF GDF-15 levels or clinical outcomes in PPMS patients after treatment with idebenone, a drug designed to alleviate mitochondrial electron-transport chain dysfunction [21]. While AHSCT is expected to prevent or at least halt mitochondrial damage caused by ongoing inflammation, its impact on pre-existing mitochondrial dysfunction related to MS is likely minimal.

YKL-40 is predominantly produced by activated astrocytes but also reflects chronic CNS inflammation [22, 23]. Elevated CSF YKL-40 levels have been associated with both acute MS exacerbations and chronic neuroinflammation, further underscoring its role in disease activity [26]. We observed a reduction in CSF YKL-40 levels over two years following AHSCT, likely indicating a general decrease in MS-related CNS inflammation. More specifically, this reduction may result from decreased activation of astrocytes and microglia. Conventional anti-inflammatory treatment for MS with powerful drugs such as natalizumab and mitoxantrone was also followed by a lowering of YKL-40 concentrations in CSF but not in serum, indicating an intrathecal inflammatory source of CSF YKL-40 [27]. We also observed a trend toward higher YKL-40 levels in patients with active disease at baseline. However, the difference was not statistically significant. This may be due to the small sample size in combination with high inter-individual variation. Only 15 patients had active disease at baseline, and their age-adjusted CSF YKL-40 levels ranged from 46.1 to 349 ng/mL. In support of this view, the two patients with post-AHSCT relapse at a sampling point both had a markedly higher CSF YKL-40 concentration at that time than at sampling points in remission before/after the relapse.

All three biomarkers, Gal-9, GDF-15, and YKL-40, are thought to reflect the status of progressive MS development. We therefore sought to determine if elevated baseline CSF concentrations of Gal-9, GDF-15, and/or YKL-40 were associated with EDA following AHSCT. We observed a trend toward slightly higher baseline levels of all three biomarkers in patients who experienced EDA during the follow-up period, although these differences were not statistically significant. The lack of significance may be attributable to limited statistical power, as the analysis included only 26 patients.

At baseline, patients treated with second-line DMDs (natalizumab or rituximab) exhibited significantly lower GDF-15 concentrations compared to treatment-naïve patients. Although specific longitudinal studies on GDF-15 modulation by second-line therapies in MS remain limited, elevated CSF GDF-15 levels have consistently been associated with progressive MS phenotypes, likely reflecting chronic neuroinflammation and mitochondrial stress [21]. Thus, reduced GDF-15 concentrations in patients on second-line therapies may indicate effective suppression of disease-related inflammation and associated neuronal stress. While our findings support the potential utility of GDF-15 as a biomarker for treatment response or disease progression, further research, including longitudinal studies, is necessary to confirm its clinical value and explore whether changes in GDF-15 correlate reliably with therapeutic efficacy.

The weakening and ultimate disappearance of correlations between Gal-9, GDF-15, YKL-40, and standard CSF measurements after AHSCT may reflect changes in the underlying pathophysiology of MS following immune system reset. Initially, higher levels of these biomarkers correlated with markers of acute inflammation and blood-CSF barrier dysfunction, suggesting close coupling between astrocytic-microglial activation (Gal-9, YKL-40), mitochondrial stress (GDF-15), and acute peripheral immune activation. However, after AHSCT, these correlations diminished progressively and were largely absent by two years, possibly indicating that the peripheral immune component and blood-brain barrier disruption play reduced roles post-treatment. This suggests that residual elevations of Gal-9, GDF-15, and YKL-40 following transplantation may reflect persistent compartmentalized neuroinflammation or neurodegeneration within the CNS [36], independent of conventional CSF inflammatory markers. Clinically, this uncoupling highlights the potential for these biomarkers to provide valuable insights into smoldering disease activity that persists despite clinical and radiological stability.

Taken together, the observed post-AHSCT decreases in CSF concentrations of Gal-9, GDF-15, and YKL-40 support the proposition that AHSCT for RRMS may slow or potentially prevent the development of progressive disease. Additionally, AHSCT has proven effective in inducing long-term remission in a high percentage of patients [3, 5, 37]. We observed that even patients with high baseline levels of Gal-9, GDF-15, and/or YKL-40 showed clinical and radiological improvement following AHSCT. Therefore, as long as progressive disease is not clinically apparent, AHSCT may offer benefits regardless of the initial levels of these biomarkers.

The most important limitation of this study is the relatively low number of patients included, especially in relation to outcomes such as post-AHSCT EDA. Another limitation is that not all patients underwent lumbar puncture at all time points, further limiting the number of available samples. The control group was not perfectly matched to the patient group in terms of age and sex, and the required age adjustments for GDF-15 and YKL-40 levels may have introduced some bias in the analysis. It is possible that a weak age correlation exists for Gal-9 as well, which we did not have statistical power to detect. However, we find it unlikely that these factors affected the main conclusion of the study.

## Conclusion

In summary, this study provides evidence that AHSCT in RRMS patients leads to a significant and sustained reduction in CSF biomarkers associated with progressive MS. These exploratory findings suggest that AHSCT may not only induce long-term remission in RRMS but also potentially influence mechanisms related to disease progression. However, given the exploratory nature of these biomarkers and the limited sample size of our study, the precise implications for delaying or preventing the onset of SPMS remain uncertain. Larger studies, preferably multicenter or randomized controlled trials, are necessary to confirm these findings and better clarify the long-term impact of AHSCT on disease progression and the predictive value of these biomarkers.

## Electronic supplementary material

Below is the link to the electronic supplementary material.

Supplementary Material 1

## Data Availability

Anonymized data are available upon reasonable request. Transfer of data will require a signed data access agreement. Requests can be sent per email to the corresponding author, Joachim Burman, joachim.burman@uu.se.

## Abbreviations

AHSCT: Autologous hematopoietic stem cell transplantation
CIS: Clinically isolated syndrome
CNS: Central nervous system
CSF: Cerebrospinal fluid
CDW: Confirmed disability worsening
DMD: Disease-modifying drug
EDA: Evidence of disease activity
EDSS: Expanded disability status scale
Gal-9: Galectin-9
GDF-15: Growth Differentiation Factor 15
IQR: Interquartile range
MNC: Mononuclear cell count
MRI: Magnetic resonance imaging
MSD: Meso scale discovery
MS: Multiple sclerosis
NEDA: No evidence of disease activity
Q_Alb_: CSF/serum albumin quotient
RRMS: Relapsing-remitting multiple sclerosis
SPMS: Secondary progressive multiple sclerosis
Th1: T-helper 1 cells
Th17: T-helper 17 cells
TIM-3: T-cell immunoglobulin mucin 3
YKL-40: Chitinase 3-like protein 1 (CHI3L1)

## References

[1] Miller AE, Chitnis T, Cohen BA, Costello K, Sicotte NL, Stacom R, et al. Autologous hematopoietic stem cell transplant in multiple sclerosis. JAMA Neurol. 2021;78(2):241. 33104165 10.1001/jamaneurol.2020.4025

[2] for the European Society for Blood and Marrow Transplantation (EBMT) Autoimmune Diseases Working Party (ADWP) and the Joint Accreditation Committee of the International Society for Cellular Therapy (ISCT) and EBMT (JACIE), Sharrack B, Saccardi R, Alexander T, Badoglio M, Burman J et al. Autologous haematopoietic stem cell transplantation and other cellular therapy in multiple sclerosis and immune-mediated neurological diseases: updated guidelines and recommendations from the EBMT Autoimmune Diseases Working Party (ADWP) and the Joint Accreditation Committee of EBMT and ISCT (JACIE). Bone Marrow Transplant. 2020;55(2):283–306. 10.1038/s41409-019-0684-0 PMC6995781 31558790

[3] Silfverberg T, Zjukovskaja C, Ljungman P, Nahimi A, Ahlstrand E, Dreimane A, et al. Haematopoietic stem cell transplantation for treatment of relapsing-remitting multiple sclerosis in sweden: an observational cohort study. J Neurol Neurosurg Psychiatry. 2024;95(2):125–33. 37748927 10.1136/jnnp-2023-331864 PMC10850659

[4] Mancardi GL, Sormani MP, Gualandi F, Saiz A, Carreras E, Merelli E, et al. Autologous hematopoietic stem cell transplantation in multiple sclerosis. Neurology. 2015;84(10):981–8. 25672923 10.1212/WNL.0000000000001329

[5] Burt RK, Balabanov R, Burman J, Sharrack B, Snowden JA, Oliveira MC, et al. Effect of nonmyeloablative hematopoietic stem cell transplantation vs continued disease-Modifying therapy on disease progression in patients with Relapsing-Remitting multiple sclerosis: A randomized clinical trial. JAMA. 2019;321(2):165–74. 30644983 10.1001/jama.2018.18743 PMC6439765

[6] Witte ME, Mahad DJ, Lassmann H, van Horssen J. Mitochondrial dysfunction contributes to neurodegeneration in multiple sclerosis. Trends Mol Med. 2014;20(3):179–87. 24369898 10.1016/j.molmed.2013.11.007

[7] Mahad DH, Trapp BD, Lassmann H. Pathological mechanisms in progressive multiple sclerosis. Lancet Neurol. 2015;14(2):183–93. 25772897 10.1016/S1474-4422(14)70256-X

[8] Barondes SH, Castronovo V, Cooper DNW, Cummings RD, Drickamer K, Felzi T, et al. Galectins: A family of animal β-galactoside-binding lectins. Cell. 1994;76(4):597–8. 8124704 10.1016/0092-8674(94)90498-7

[9] Wada J, Kanwar YS. Identification and characterization of galectin-9, a novel β-galactoside-binding mammalian lectin. J Biol Chem. 1997;272(9):6078–86. 9038233 10.1074/jbc.272.9.6078

[10] Seki M, Oomizu S, Sakata K-M, Sakata A, Arikawa T, Watanabe K, et al. Galectin-9 suppresses the generation of Th17, promotes the induction of regulatory T cells, and regulates experimental autoimmune arthritis. Clin Immunol. 2008;127(1):78–88. 18282810 10.1016/j.clim.2008.01.006

[11] Zhu C, Anderson AC, Schubart A, Xiong H, Imitola J, Khoury SJ, et al. The Tim-3 ligand galectin-9 negatively regulates T helper type 1 immunity. Nat Immunol. 2005;6(12):1245–52. 16286920 10.1038/ni1271

[12] Oomizu S, Arikawa T, Niki T, Kadowaki T, Ueno M, Nishi N, et al. Cell surface galectin-9 expressing Th cells regulate Th17 and Foxp3 + Treg development by galectin-9 secretion. PLoS ONE. 2012;7(11):e48574. 23144904 10.1371/journal.pone.0048574 PMC3492452

[13] Anderson AC, Anderson DE, Bregoli L, Hastings WD, Kassam N, Lei C, et al. Promotion of tissue inflammation by the immune receptor Tim-3 expressed on innate immune cells. Science. 2007;318(5853):1141–3. 18006747 10.1126/science.1148536

[14] Steelman AJ, Li J. Astrocyte galectin-9 potentiates microglial TNF secretion. J Neuroinflammation [Internet]. 2014;11(1). Available from: 10.1186/s12974-014-0144-0 10.1186/s12974-014-0144-0 PMC4158089 25158758

[15] Steelman AJ, Smith R 3rd, Welsh CJ, Li J. Galectin-9 protein is up-regulated in astrocytes by tumor necrosis factor and promotes encephalitogenic T-cell apoptosis. J Biol Chem. 2013;288(33):23776–87. 23836896 10.1074/jbc.M113.451658 PMC3745324

[16] Burman J, Svenningsson A. Cerebrospinal fluid concentration of Galectin-9 is increased in secondary progressive multiple sclerosis. J Neuroimmunol. 2016;292:40–4. 26943957 10.1016/j.jneuroim.2016.01.008

[17] Bootcov MR, Bauskin AR, Valenzuela SM, Moore AG, Bansal M, He XY, et al. MIC-1, a novel macrophage inhibitory cytokine, is a divergent member of the TGF-β superfamily. Proc Natl Acad Sci U S A. 1997;94(21):11514–9. 9326641 10.1073/pnas.94.21.11514 PMC23523

[18] Breit SN, Johnen H, Cook AD, Tsai VWW, Mohammad MG, Kuffner T, et al. The TGF-β superfamily cytokine, MIC-1/GDF15: A pleotrophic cytokine with roles in inflammation, cancer and metabolism. Growth Factors. 2011;29(5):187–95. 21831009 10.3109/08977194.2011.607137

[19] Desmedt S, Desmedt V, De Vos L, Delanghe JR, Speeckaert R, Speeckaert MM. Growth differentiation factor 15: A novel biomarker with high clinical potential. Crit Rev Clin Lab Sci. 2019;56(5):333–50. 31076013 10.1080/10408363.2019.1615034

[20] Xue X-H, Tao L-L, Su D-Q, Guo C-J, Liu H. Diagnostic utility of GDF15 in neurodegenerative diseases: A systematic review and meta-analysis. Brain Behav. 2022;12(2):e2502. 35068064 10.1002/brb3.2502 PMC8865151

[21] Kosa P, Wu T, Phillips J, Leinonen M, Masvekar R, Komori M, et al. Idebenone does not inhibit disability progression in primary progressive MS. Mult Scler Relat Disord. 2020;45(102434):102434. 32784117 10.1016/j.msard.2020.102434 PMC9386688

[22] Bonneh-Barkay D, Wang G, Starkey A, Hamilton RL, Wiley CA. In vivo CHI3L1 (YKL-40) expression in astrocytes in acute and chronic neurological diseases. J Neuroinflammation. 2010;7(1):34. 20540736 10.1186/1742-2094-7-34 PMC2892443

[23] Cantó E, Tintoré M, Villar LM, Costa C, Nurtdinov R, Álvarez-Cermeño JC, et al. Chitinase 3-like 1: prognostic biomarker in clinically isolated syndromes. Brain. 2015;138(4):918–31. 25688078 10.1093/brain/awv017

[24] Momtazmanesh S, Shobeiri P, Saghazadeh A, Teunissen CE, Burman J, Szalardy L, et al. Neuronal and glial CSF biomarkers in multiple sclerosis: a systematic review and meta-analysis. Rev Neurosci. 2021;32(6):573–95. 33594840 10.1515/revneuro-2020-0145

[25] Mañé-Martínez MA, Olsson B, Bau L, Matas E, Cobo-Calvo Á, Andreasson U, et al. Glial and neuronal markers in cerebrospinal fluid in different types of multiple sclerosis. J Neuroimmunol. 2016;299:112–7. 27725108 10.1016/j.jneuroim.2016.08.004

[26] Burman J, Raininko R, Blennow K, Zetterberg H, Axelsson M, Malmeström C. YKL-40 is a CSF biomarker of intrathecal inflammation in secondary progressive multiple sclerosis. J Neuroimmunol. 2016;292:52–7. 26943959 10.1016/j.jneuroim.2016.01.013

[27] Malmeström C, Axelsson M, Lycke J, Zetterberg H, Blennow K, Olsson B. CSF levels of YKL-40 are increased in MS and decrease with immunosuppressive treatment. J Neuroimmunol. 2014;269(1–2):87–9. 24582001 10.1016/j.jneuroim.2014.02.004

[28] Thompson AJ, Banwell BL, Barkhof F, Carroll WM, Coetzee T, Comi G, et al. Diagnosis of multiple sclerosis: 2017 revisions of the McDonald criteria. Lancet Neurol. 2018;17(2):162–73. 29275977 10.1016/S1474-4422(17)30470-2

[29] Teunissen CE, Petzold A, Bennett JL, Berven FS, Brundin L, Comabella M, et al. A consensus protocol for the standardization of cerebrospinal fluid collection and biobanking. Neurology. 2009;73(22):1914–22. 19949037 10.1212/WNL.0b013e3181c47cc2 PMC2839806

[30] Herman S, Khoonsari PE, Tolf A, Steinmetz J, Zetterberg H, Åkerfeldt T, et al. Integration of magnetic resonance imaging and protein and metabolite CSF measurements to enable early diagnosis of secondary progressive multiple sclerosis. Theranostics. 2018;8(16):4477–90. 30214633 10.7150/thno.26249 PMC6134925

[31] TNF neutralization in MS. Results of a randomized, placebo-controlled multicenter study. Neurology. 1999;53(3):457–457. 10449104

[32] Correale J, Farez MF. The role of astrocytes in multiple sclerosis progression. Front Neurol. 2015;6:180. 26347709 10.3389/fneur.2015.00180 PMC4539519

[33] Montero R, Yubero D, Villarroya J, Henares D, Jou C, Rodríguez MA, et al. GDF-15 is elevated in children with mitochondrial diseases and is induced by mitochondrial dysfunction. PLoS ONE. 2016;11(2):e0148709. 26867126 10.1371/journal.pone.0148709 PMC4750949

[34] Yatsuga S, Fujita Y, Ishii A, Fukumoto Y, Arahata H, Kakuma T, et al. Growth differentiation factor 15 as a useful biomarker for mitochondrial disorders. Ann Neurol. 2015;78(5):814–23. 26463265 10.1002/ana.24506 PMC5057301

[35] Poulsen NS, Madsen KL, Hornsyld TM, Eisum A-SV, Fornander F, Buch AE, et al. Growth and differentiation factor 15 as a biomarker for mitochondrial myopathy. Mitochondrion. 2020;50:35–41. 31669236 10.1016/j.mito.2019.10.005

[36] Cross AH, Gelfand JM, Thebault S, Bennett JL, von Büdingen HC, Cameron B et al. Emerging cerebrospinal fluid biomarkers of disease activity and progression in multiple sclerosis. JAMA Neurol [Internet]. 2024; Available from: 10.1001/jamaneurol.2024.0017 10.1001/jamaneurol.2024.0017 PMC10928543 38466277

[37] Boffa G, Massacesi L, Inglese M, Mariottini A, Capobianco M, Moiola L, et al. Long-term clinical outcomes of hematopoietic stem cell transplantation in multiple sclerosis. Neurology. 2021;96(8):e1215–26. 33472915 10.1212/WNL.0000000000011461

