# Supplementary material for: Biomarkers of progressive multiple sclerosis decrease following autologous hematopoietic stem cell transplantation
Source: J Neuroinflammation. 2025 Jul 17;22:186. doi: 10.1186/s12974-025-03511-6 (PMC12273295; doi:10.1186/s12974-025-03511-6)
Supplement: Supplementary file 1 — Supplementary Material 1 [file 12974_2025_3511_MOESM1_ESM.pdf]

Supplementary data - Biomarkers of progressive multiple sclerosis are decreased after hematopoietic stem cell transplantation

Supplementary Figure S1. – At-baseline biomarker levels and treatment with DMDs.

Supplementary Figure S2 - Baseline biomarker level comparison between patients with and patients without disease activity post-AHSCT.

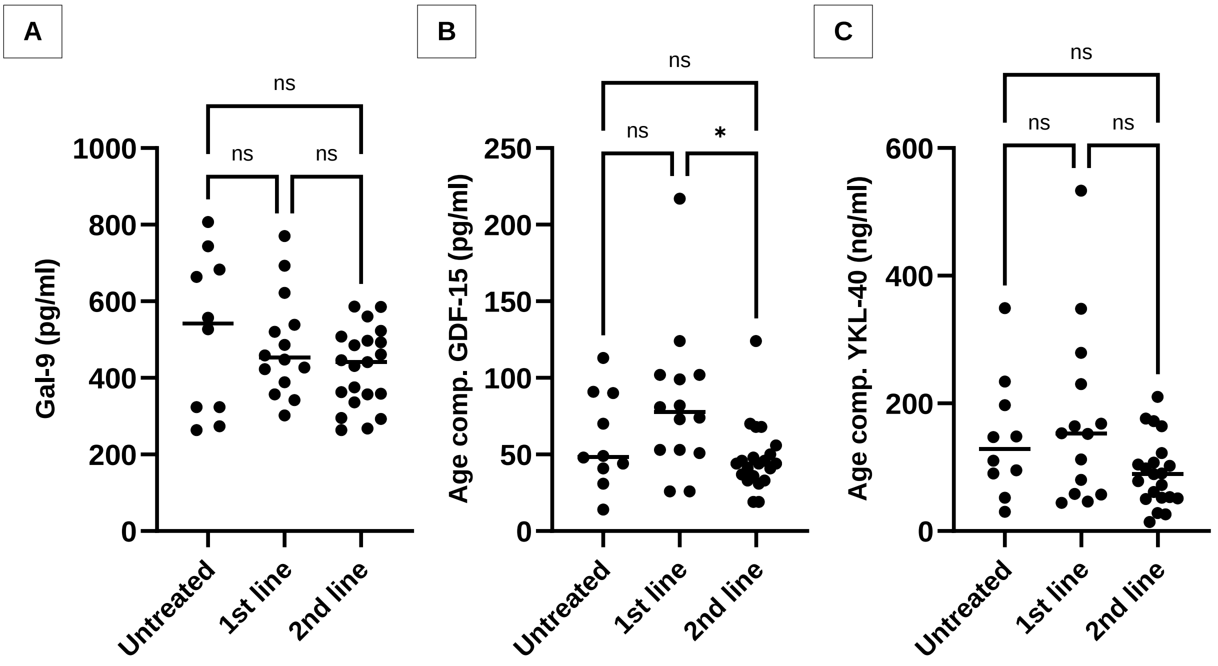

Supplementary Figure S1. At-baseline biomarker levels and treatment with DMDs.

At-baseline concentrations of Gal-9 (A), GDF-15 (B) and YKL-40 (C) in cerebrospinal fluid from untreated vs 1st line DMD treated vs 2nd line DMD treated RRMS patients. ns = non significant, * $p \leq 0.05$.

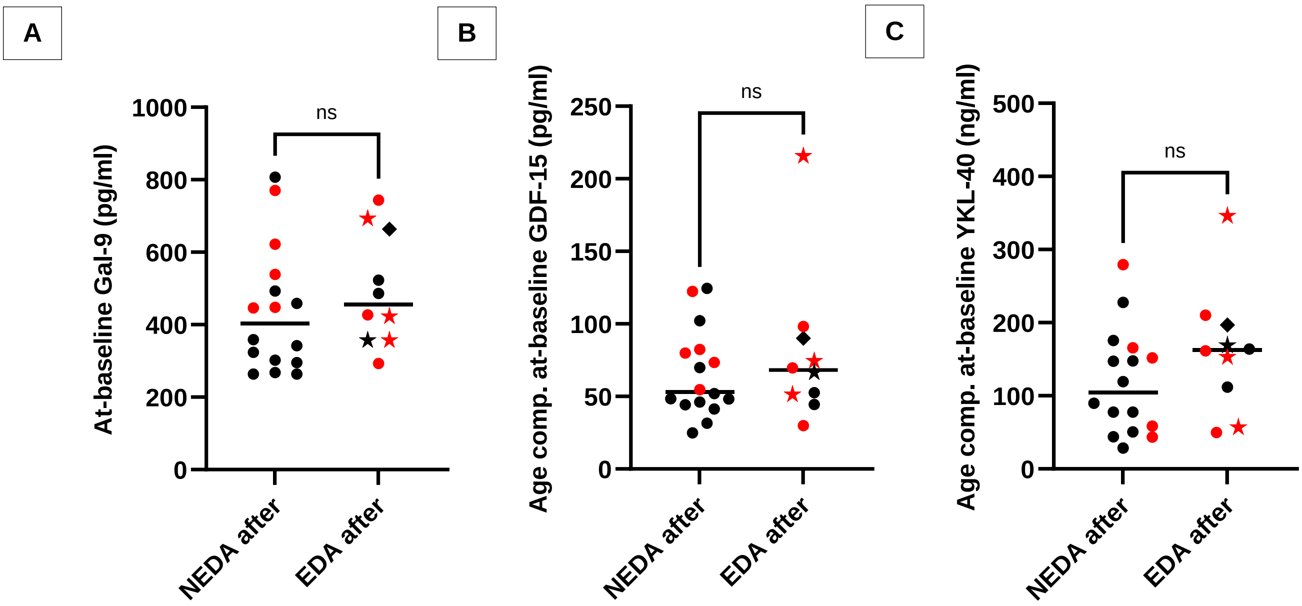

Supplementary Figure S2. Baseline biomarker level comparison between patients with and patients without post-AHSCT disease activity

At-baseline Gal-9 (A), GDF-15 (B), and YKL-40 (D) concentrations in cerebrospinal fluid of those 26 patients who have been followed for ≥ 4 years comparing patients with and patients without post-AHSCT disease activity. NEDA after = no evidence of disease activity *after AHSCT*. EDA after = evidence of disease activity *after AHSCT*. The horizontal line shows the median of the group. Red dot = active disease *at baseline*. Star = clinical relapse after AHSCT. Rhomb = confirmed disability worsening without MRI activity. ns = not significant.
